# Preservation of Chocolate Muffins with Lemon Balm, Oregano, and Rosemary Extracts

**DOI:** 10.3390/foods10010165

**Published:** 2021-01-15

**Authors:** Mariana C. Pedrosa, Jonata M. Ueda, Bruno Melgar, Maria Inês Dias, José Pinela, Ricardo C. Calhelha, Marija Ivanov, Marina Soković, Sandrina Heleno, Aline Bruna da Silva, Márcio Carocho, Isabel C. F. R. Ferreira, Lillian Barros

**Affiliations:** 1 Centro de Investigação de Montanha (CIMO), Instituto Politécnico de Bragança, Campus de Santa Apolónia, 5300-253 Bragança, Portugal; marianapedrosa@ipb.pt (M.C.P.); massaoueda@hotmail.com (J.M.U.); bruno.melgarc@ipb.pt (B.M.); maria.ines@ipb.pt (M.I.D.); jpinela@ipb.pt (J.P.); calhelha@ipb.pt (R.C.C.); sheleno@ipb.pt (S.H.); iferreira@ipb.pt (I.C.F.R.F.); 2 Institute for Biological Research “Siniša Stanković”—National Institute of Republic of Serbia, University of Belgrade, Blvd. despot Stefan 142, 11000 Belgrade, Serbia; marija.smiljkovic@ibiss.bg.ac.rs (M.I.); mris@ibiss.bg.ac.rs (M.S.); 3 Department of Materials Engineering, Centro Federal de Educação Tecnológica de Minas Gerais, Av. Amazonas, 5253, Belo Horizonte, MG 30421-169, Brazil; alinebruna@cefetmg.br

**Keywords:** chocolate muffins, food incorporation, natural preservatives

## Abstract

Muffins are snacks made from flour and chocolate and preserved with synthetic additives. Following consumer trends, the search for natural food additives has gained traction. Plants such as rosemary, lemon balm, and oregano were analyzed following an optimization of ultrasound assisted extraction, screened for their antioxidant and antimicrobial activity and incorporated in chocolate muffins, comparing them to synthetic preservatives over the course of 8 days. The nutritional profile, organic and fatty acids, soluble sugars, texture profile, external color and digital imaging of the muffin pores were analyzed. Slight changes were sought for the muffins incorporated with the natural extracts. By means of linear discriminant analysis, rosemary extract was considered the most promising extract to preserve the muffins due to its similarity to potassium sorbate, showing no changes in the muffins it was incorporated in, although it showed a lower amount of phenolic compounds when compared to lemon balm.

## 1. Introduction

One of the main sources of nutrients for human beings are cereal grains such as wheat, rice, corn starch, sorghum and barley. The energy present in cereal grains is an important contribution to the diet, as are proteins, oils and vitamins, although they are present in smaller quantities. In Europe, wheat and baked products made of flour are historically the basis of the diet [1]. A muffin is a wheat product, sweet, highly caloric and very popular, which is baked and usually consumed throughout the day. In recent years, it has gained the status of a “snack” and it is now consumed around the world [2,3,4,5,6]. One of the most important aspects in the development of food is its preservation, which relies, nowadays, on synthetic or artificial food additives. The use of additives is a technique which aims at reducing or inhibiting chemical and microbial deterioration, therefore, food waste and loss [7,8]. The tendency to use natural additives is due to the fact that consumers perceive them as not having the harmful effects on health that some synthetic ones may have, including toxicity, allergic reactions, or the risk of a daily over-exposure [9]. In order to meet these consumer demands for safer, healthier, differentiated and functional foods, the scientific community and the food industry itself have been intensifying the search for innovations and alternatives, for example, by using plant extracts to replace artificial additives [10,11,12]. Several studies have indicated that the Lamiaceae family of plants has a great variety of molecules, with biological activity and great number of different phenolic compounds, being powerful natural antioxidants to combat oxidative “stress” and, consequently, metabolic and pathological disturbances [13,14,15]. Thus, the objective of this work was to optimize ultrasonic extractions of rosemary (*Rosmarinus officinalis* L.), lemon balm (*Melissa officinalis* L.) and oregano (*Origanum vulgare* L.), characterize the extracts in terms of their chemical components and bioactivities, and finally use them as preservative ingredients in chocolate muffins, comparing them to synthetic preservatives (sodium benzoate (E211) and potassium metabisulfite (E224)) over 8 days.

## 2. Materials and Methods

### 2.1. Chemicals, Reagents and Samples

All chemicals and reagents were acquired from scientific retailers, and were of, at least, analysis purity, unless when used for high-performance liquid chromatography (HPLC), when they were of HPLC grade. The plant samples were acquired from “Cantinho das Aromáticas”, an enterprise located in Vila Nova de Gaia, Portugal, that commercializes aromatic and medicinal plants.

### 2.2. Plant Samples

The plants, rosemary (*Rosmarinus officinalis* L.), lemon balm (*Melissa officinalis* L.) and oregano (*Origanum vulgare* L.), were acquired from the Portuguese enterprise “Cantinho das Aromáticas” based in Vila Nova de Gaia, in a dried state, and thus, immediately after arrival at the lab, they were ground to a fine powder of about 20 mesh and stored away from light in a cool and dry place until analysis.

### 2.3. Optimization of Ultrasound Assisted Extraction

The plants were subject to two kinds of extractions, namely ultrasound assisted extraction (UAE) with an ultrasonic probe system (CY-500, Optic Ivymen Systems, Barcelona, Spain) and a heat-assisted maceration, followed by an optimization process to determine the ideal extraction process and the optimal conditions to obtain higher yields in rosmarinic acid, the most bioactive compound usually present in these samples.

#### 2.3.1. Ultrasound Assisted Extraction

The UAE used three variables for optimization, namely extraction time, ultrasonic potency and finally solvent percentage, in which the varying solvent was ethanol, with the base solvent being distilled water. The plants were mixed in the different solutions at a concentration of 25 g/L and were subjected to ultrasonic extraction using values within the ranges detailed above. After each extraction the solutions were centrifuged (5000 rpm for 20 min at 10 °C), filtered through a Nº4 Whatman paper and, when necessary, ethanol was removed through a rotary evaporator and subsequently freeze-dried (FeeeZone 4.5, Labconco, Kansas City, MO, USA).

#### 2.3.2. Optimization Procedures

Initially, a fractional factorial design was used to optimize the extraction of rosmarinic acid in the rosemary extracts using a normal two-level factorial design with resolution III (2^(3−1)^) using the Design Expert software (Stat-Ease, Inc., Minneapolis, MN, USA). This design was composed of 4 runs with the fixed variables varying between X1—time (7.5 to 12.5 min), X2—solvent (0 to 80% ethanol) and X3—ultrasonic potency (275 to 450 watts), with the dependent variable being the amount of rosmarinic acid (Y1). The factorial effects alias was built as Intercept = Intercept + ABC, A = A + BC, B = B + AC and C = C + AB. Then, a general factorial design was used to narrow down the optimal conditions, initially fixing the potency at 55% and solvent percentage at 80, varying time from 10 s to 5 min (a lower amount than the fractional factorial design). The final and third stage fixed all three parameters and varied temperature (X4) between 20 and 75 °C, allowing us to obtain the parameters that yield the maximum amount of rosmarinic acid.

#### 2.3.3. Phenolic Profile

The same high-performance liquid chromatography (HPLC) equipment was used for the quantification of rosmarinic acid for the optimization of the extraction and the quantification of the phenolic profile of the extracts at the optimized parameters. The system consisted of a Dionex Ultimate 3000 HPLC (Thermo Scientific, San Jose, CA, USA), coupled to a diode array detector (DAD, set at 280, 330, and 370 nm) and a mass detector (MS), and also equipped with a quaternary pump, an automated injector (5 °C), a degasser, and a temperature-adjusted column chamber (35 °C) [16]. The detection of compounds was achieved through a diode array detector (DAD) set at wavelengths of 280, 330 and 370 nm and coupled to a mass detector (MS). The separation of the compounds relied on a C18 columns (Waters Spherisorb S3 ODS-2 (3 µm, 4.6 mm × 150 mm, Waters, Milford, MA, USA) operating at 35 °C and the mobile phase consisted of 0.1% of formic acid in water and acetonitrile, functioning in gradient mode. The gradient varied from 15% of acetonitrile for 5 min to 20% for another 5 min, then 10 min at 25%, another 10 min to 35% and finally another 10 min from 35 to 50%, with the column being rebalanced from 10 min at a flux of 0.5 mL/min. The detection of masses was achieved using a Liner LTQ XL ion trap mass spectrometer (Thermo Finnigan, San Jose, CA, USA) equipped with an electrospray ionization source using nitrogen as the carrier gas [16]. The data were analyzed using Xcalibur software and the compounds were identified according to their retention times, UV-Vis spectra, and mass fragmentation compared to standard compounds (when available) and/or using data available in the literature. For the quantification of each compound, 7-level calibration curves were used (Extrasynthèse, Genay, France): cinnamic acid (y = 1 × 10^−6^ − 222,204, R^2^ = 0.9993, LOD = 0.12 µg/mL and LOQ = 0.83 µg/mL); quercetin-3-*O*-glucoside (y = 34,843x − 160,173; R2 = 1.000; LOD = 0.21 µg/mL and LOQ = 0.71 µg/mL); rosmarinic acid (y = 191,291x − 652,903; R^2^ = 0.999; LOD = 0.15 µg/mL and LOQ = 0.68 µg/mL). The compounds that could not be quantified by their specific standards were quantified using calibration curves from the most related available standard compounds. The results were expressed in mg/g of extract.

### 2.4. Bioactive and Cytotoxic Analysis

Antimicrobial activity: the antibacterial activity of the extracts was evaluated by the microplate microdilution method. The minimum inhibitory concentrations (MIC) of the bacterial species were determined as described by [17] using three Gram-positive bacteria, *Staphylococcus aureus* (ATCC 11632), *Bacillus cereus* (clinical isolate), *Listeria monocytogenes* (NCTC 7973), as well as Gram-negative bacteria, *Escherichia coli* (ATCC 25922), *Salmonella typhimurium* (ATCC 13311) and *Enterobacter cloacae* (ATCC 35030). For comparison’s sake, two artificial food preservatives usually applied in this type of snack were used (E211, E224). Streptomycin and ampicillin were used as positive controls. For the antifungal assays, the MICs of six micromycetes were calculated: *Aspergillus fumigatus* (human isolate), *Aspergillus niger* (ATCC 6275), *Aspergillus versicolor* (ATCC11730), *Penicillium funiculosum* (ATCC 36839), *Trichoderma viride* (IAM 5061) and *Penicillium verrucosum* var. cyclopium (food isolate). Ketoconazole and bifonazole were used as positive controls, as well as the two artificial food preservatives stated above. The MICs were calculated after adding 40 μL of *p*-iodonitrotetrazolium chloride at 0.2 mg/mL, dissolved in the water of the microplate wells and incubated at 37 °C for 10–30 min.

Antioxidant activity: the antioxidant activity was determined by the oxidative hemolysis inhibition assay (OxHLIA), according to [18]. A sheep red blood cell solution (2.8%, *v*/*v*; 200 µL) prepared in phosphate-buffered saline (PBS) was mixed with 400 µL of the extracts (6–500 μg/mL in PBS), PBS (control), water (for complete hemolysis), or the positive control trolox (7.81–250 µg/mL PBS). After pre-incubation at 37 °C for 10 min with shaking, 200 μL of 2,2′-azobis(2-methylpropionamidine) dihydrochloride (AAPH, 160 mM in PBS) were added and the optical density was measured at 690 nm every ~10 min in a microplate reader (Bio-Tek Instruments, ELX800) until complete hemolysis. The *Δt* values (min) resulting from the half hemolysis time (H*t*_50_ values) obtained from the hemolytic curves of each extract sample concentration minus the H*t*_50_ value of the PBS control were correlated to the respective extract concentration to obtain IC_50_ values (µg/mL), which were calculated for time periods of 120 and 180 min, i.e., extract concentration required to protect 50% of the red blood cell population from the hemolytic action of AAPH for 120 and 180 min.

Cytotoxicity: to screen the safety of the obtained extracts, they were tested for their toxicity for normal cells (porcine liver primary culture PLP2), by the sulphorrodamine B assay, as described by [18]. The extracts were analyzed in a range of concentrations from 400 to 1.56 μg/mL allowing the determination of the extract’s concentration providing 50% of cell growth inhibition (GI50, µg/mL). Ellipticin was used as a positive control.

### 2.5. Muffin Preparation

The muffins were prepared following the recipe described by [19], with some changes, which included, for 9 muffins, flour (100 g), eggs (56 g), powdered milk (50 g), sugar (60 g), powdered chocolate (15 g), vegetable oil (35 g), baking powder (5 g), citric acid (0.75 g), salt (1.25 g) and water (35 g). Nine batches of nine muffins (for each time stamp) were produced, varying the type of preservative and extract used, with one of the batches being the control, without the addition of any preservative. After weighing the quantity of each ingredient, the dry ingredients were added (except the baking powder and the extract), mixed for 20 s. Then the oil and water (with the dissolved preservative) were added together along with the baking powder. The oven was preheated, and the muffins were baked in a convective oven in a muffin tray for 20 min at 180 °C with subsequent cooling for 10 min. The first batch (T0) was analyzed immediately. The other batches were stored in sealed plastic containers and analyzed after 4 and 8 days. The quantities of the preservatives added to each muffin was based on the recommended quantity allowed by legislation within the European Union for the approved natural preservative rosemary extract (E392–200 mg/kg fat) in bakery products. Furthermore, in other muffins, the legal maximum amount of potassium sorbate added to pastry snacks (2 mg/g) was used for the natural extracts and potassium sorbate itself.

### 2.6. Nutritional and Chemical Characterization

The analyses of the muffins included the nutritional profile, and the chromatographic analysis of organic acids, soluble sugars and fatty acids.

#### 2.6.1. Nutritional Profile

The nutritional profile of the muffins was analyzed following the official AOAC methodology, 17th Ed. [20]. The moisture content was analyzed following AOAC method 925.09, in which 2 g of the sample were placed in a metal dish which was closed and weighed. The dish was placed in an oven (Scientific Series, Contherm, New Zealand) at 100 °C for 5 h, and after cooling down was weighed once again. The moisture was calculated by subtracting the final weight to the initial one. Total available carbohydrates were calculated by difference from all other nutrients. The dietary fiber was calculated using the AOAC procedure 993.19, through the enzymatic–gravimetric method, in which the sample is degraded with α-amylase, protease and amyloglucosidase prior to being filtered through crucibles with celite. Crude protein content was calculated relying on the Macro Kjedahl method, following the AOAC 920.87 method, using a conversion factor of 6.25. In the process, 0.5 g of the samples were digested in K_2_SO_4_/CuSO_4_ catalyst and sulfuric acid at 400 °C for 70 min. Then, an integrated alkaline stead distillation and titration took place in a Kjeldahl distiller (model Pro-Nitro-A, JP Selecta, Barcelona). Crude fat was calculated following an extraction. A Soxhlet apparatus was used to extract and quantify the crude fat, using 3 g of sample and petroleum ether as an extracting solvent. Total mineral content was calculated following the AOAC 923.03, in which 0.5 g of the sample was incinerated in a muffle (Lenton ECF 12/22, Hope Valley, UK) at 550 °C. All nutrients were expressed as g/100 g of fresh weight (fw). To calculate the energy, the following formula, based on the European Parliament and Council Regulation No. 1169/2011, was used:

Energy = 4 × (g protein + g carbohydrates) + 2 × (g dietary fibre) + 9 × (crude fat)(1)

#### 2.6.2. Organic Acids Analysis

Organic acids were evaluated using ultra-fast liquid chromatography (UFLC, Shimadzu 20A series, Kyoto, Japan) coupled to a diode array detector, as previously defined by [21]. Organic acids standards (L(+)-ascorbic acid, citric acid, malic acid, oxalic acid, shikimic acid, succinic acid, and quinic acid, were used for identification by performing chromatographic comparisons with the peaks of the samples. These standards were also used for quantification, relying on the external standard methodology. Results were expressed as g/100 g fw.

#### 2.6.3. Soluble Sugars Analysis

The soluble sugars were determined through (HPLC) coupled to a refraction index (RI) detector. The procedure followed the one previously reported by [21], using melezitose as the internal standard. The equipment consisted of a pump and degasser (Knauer, Smartline system 1000, Berlin, Germany), and an auto sampler (AS-2057 Jasco, Easton, MD, USA), coupled to a refraction index detector (Knauer). Sugars were identified by comparing their peaks to the retention times of commercial standards, with the data being analyzed with the Clarity 2.4 software (DataApex, Prague, Czech Republic). Results were expressed as g/100 g fw.

#### 2.6.4. Individual Fatty Acids Analysis

Fatty acids were determined by gas chromatography (GC) coupled to a flame ionization detector (FID). The equipment consisted of a DANI GC (DANI 1000, Contone, Switzerland) with a split/splitless injector. Briefly, the fat was extracted with petroleum ether in a Soxhlet apparatus, then methylated with 5 mL of a solution of methanol:sulphuric acid:toluene (2:1:1) overnight at 50 °C and 160 rpm. Then, 3 mL of deionized water was added for phase separation and the fatty acid methyl esters were recovered to vials and injected into a GC-FID system. The column used was a Zebron-Kame (30 m × 0.25 mm i.d., 0.20 µm). The oven temperature followed the pattern: starting temperature of 100 °C, held for 2 min, then, a ramp of 10 °C/min to 140 °C, followed by a 3 °C/min to 190 °C, 30 °C/min ramp to 260 °C held for 2 min. The carrier gas (hydrogen) was maintained at 1.1 mL/min (0.61 bar), measured at 100 °C. Split injection (1:50) was performed at 250 °C, and the identification of the individual fatty acids was achieved by comparing the retention times of the fatty acid methyl esters to commercial standards, namely FAME Mix C4-C24 (standard 4788-U, Sigma-Aldrich). The same software used for the soluble sugars was employed for the fatty acids. The results were presented as a relative percentage of each fatty acid.

### 2.7. Physical Analyses

The physical analyses encompassed the external color, texture and digital imaging.

#### 2.7.1. External Color

The external color was measured in three different points of the top of the muffins, and also another three on their side. This assay was performed with a portable CR400 colorimeter from Konica Minolta (Chiyoda, Tokyo, Japan) with the D65 illuminant, a standard illuminant defined by the International Commission on Illumination (CIE) which represents midday light in Europe (daylight illuminant). The CIE L* a* b* color space of 1976 was used, with L* representing lightness, a* representing redness (red-green), and b* representing yellowness (yellow-blue), with a 10° observation angle and 8 mm aperture.

#### 2.7.2. Texture Profile

A Stable Micro Systems (Vienna Court, Godalming UK TA.XT) Plus Texture Analyzer with a 30 Kg load cell, using the P/35 35 mm aluminum cylinder probe was used to carry out the texture analysis. Texture profile analysis (TPA) was performed on the samples using 5 mm/s as the pre- and post-test speed, and 3 mm/s as the test speed. The target mode was set to “strain” at 25% strain level for 5 consecutive seconds, while the trigger was set to “force” with measurement starting at 10 g of force. After the analysis, a macro was used to measure various dimensions of texture, namely hardness, adhesiveness, springiness, cohesiveness, chewiness and resilience. The texture results were reached through the Exponent program, proprietary of Stable Micro Systems.

#### 2.7.3. Digital Imaging

The digital imaging followed the procedure of [22] and the theory of [23] with slight modifications. Images of three slices were obtained from a flatbed scanner (Canon PIXMA, MG 2550). The brightness and contrast of the images were both set to zero, and the images were converted to bitmap files with a resolution of 300 dpi and 40 × 40 mm square field of view, and finally saved in black and white using Adobe Photoshop 2020. Using the ImageJ-based Fiji 1.46 software package, the image was pre-processed and converted to 8-bit greyscale and subsequently subject to auto threshold using the Otsu algorithm before using the object counting function to return the number of objects, average size, and circularity.

### 2.8. Statistical Tools

Throughout the manuscript, all data are expressed as mean ± standard deviation (SD). The analyses performed on the muffins were interpreted through two-way analysis of variance (ANOVA) with type III sums of squares, after verifying homoscedasticity through a Levene’s test. The post hoc test used was either a Tukey’s multiple test (homoscedastic samples) or Tamhane T2 test (non-homoscedastic samples). Using two-way ANOVA, it is possible to verify the influence of the two factors-storage time (ST) and type of preservative (TP). If a significant interaction was detected among the two factors (ST × TP *p* < 0.05), they were evaluated simultaneously, and some tendencies were extracted from the estimated marginal means plots (EMM). Inversely, if there was no significant interaction recorded among the two factors (ST × TP *p* > 0.05), they were analyzed and classified independently using the post hoc tests described above. Thus, the SDs were calculated from results obtained under different operational conditions, and should, therefore not be regarded as a measure of precision, rather as the range of the recorded values. All statistical analysis was performed using a significance of 0.05, using Design Expert and the SPSS software, version 25. A linear discriminant analysis (LDA) was performed to discriminate the different preservatives and the three storage times. The LDA used Wilk’s λ test with an *F*-value of 3.84 for entering and 2.71 for removal, using the leave-one-out cross validation procedure.

## 3. Results and Discussion

### 3.1. Optimization of Rosmarinic Acid Extraction

From the studied parameters (time of extraction, ultrasonic power, and solvent percentage), the one with the highest influence was the amount of solvent, with a two-fold increase in magnitude when compared with the effect of time (second most important factor). Thus, the ultrasonic power was set at 375 W and a multilevel factorial design was adopted to further optimize the other two parameters. The use of time intervals between 7.5 and 12.5 min proved to not have a significant effect, and thus, an interval between 10 s and 5 min was tested, showing that smaller periods of extraction did not seem to reduce the extraction efficiency. The four most abundant phenolic compounds were identified and quantified, and included phenolic acids and flavonoids The peaks were tentatively identified according to their chromatographic responses regarding their retention time, UV-Vis spectra, and mass fragmentation. For the phenolic acids group, two compounds were found. Peak **4** was identified as rosmarinic acid ([M-H]^−^ at *m/z* 359), by comparison its retention time and UV-vis spectra with the corresponding available standard compound. This compound has been previously identified in the infusion extracts of in vitro cultured and commercial samples of lemon balm by [24]; in the aqueous and hydroalcoholic extracts of oregano by [25]; and also, in hydroalcoholic extracts of rosemary by [26]. The second phenolic acid found was peak **1**, that presented a pseudomolecular ion at [M-H]^−^ at *m/z* 421, and MS^2^ fragment at *m/z* 315 (106 u), *m/z* 299 (122 u), *m/z* 259 (162 u), and *m/z* 153 (106 u), that are coherent with the previously described by [27] in the leaves of *Ilex glabra* L. Gray., being tentatively identified as 4-hydroxy-7-*O*-(3′hydroxy-4′-*O*-glucosylbenzyl)benzyl. To the authors′ knowledge, this is the first time that this compound has been reported in lemon balm, oregano, and rosemary samples.

Regarding the group of flavonoids, two peaks were also identified in the studied samples. Peaks **2** and **3**, presented the same pseudomolecular ion [M-H]^−^ at *m/z* 461 and the same unique MS^2^ fragment at *m/z* 285, that corresponded to the loss of a glucuronyl moiety. The presence of the aglycone at *m/z* 285 could be identified as kaempferol or luteolin, being differentiated through its UV-Vis spectra. In this manner, the identification of peak **2** showed a UV-Vis spectra characteristic to a luteolin aglycone and peak **3** to a kaempherol moiety, being tentatively identified as luteolin-*O*-glucuronide and kaempferol-*O*-glucuronide, respectively.

Lemon balm showed the highest amount of total phenolic compounds as well as the highest amount of rosmarinic acid. The choice of using all three extracts for incorporation in the muffins helps understand the effect of rosmarinic acid as the main bioactive compound, or if the bioactive potential can be due to synergistic effects between the different compounds.

The final parameter to analyze was the effect of temperature, with a variation of 20 to 75 °C, to understand if any of the major compounds were thermolabile. For rosemary, a negative effect was associated with the increase in temperature, showing that at 20 °C the yield was 40% higher when compared to 75 °C. Lemon balm, on the contrary, showed a positive trend at higher temperatures, as well as oregano, although for this plant the temperature did not show much difference. In this manner, the optimal conditions for rosemary were 0.25 min, 375 W, 80% ethanol at 20 °C. The optimal conditions for lemon balm were set at 2 min of extraction, 375 W at 80% of ethanol and 75 °C. Finally, oregano had the highest rosmarinic acid extractability at 2 min of extraction, 375 W, 80% ethanol and 75 °C. These results are in agreement with [28], who reported that adding ethanol to an ultrasonic extraction increases the yield of extract for rosemary. Furthermore, it is also in agreement with [29], in which lemon balm’s extraction yield is increased with temperature.

### 3.2. Bioactive and Cytotoxic Evaluation of the Extracts

After obtaining the optimal conditions, the dried extracts were freeze-dried and subjected to antimicrobial, antioxidant and cytotoxic assays to understand their bioactivity and toxicity to non-tumoral cells.

**Figure 1:**
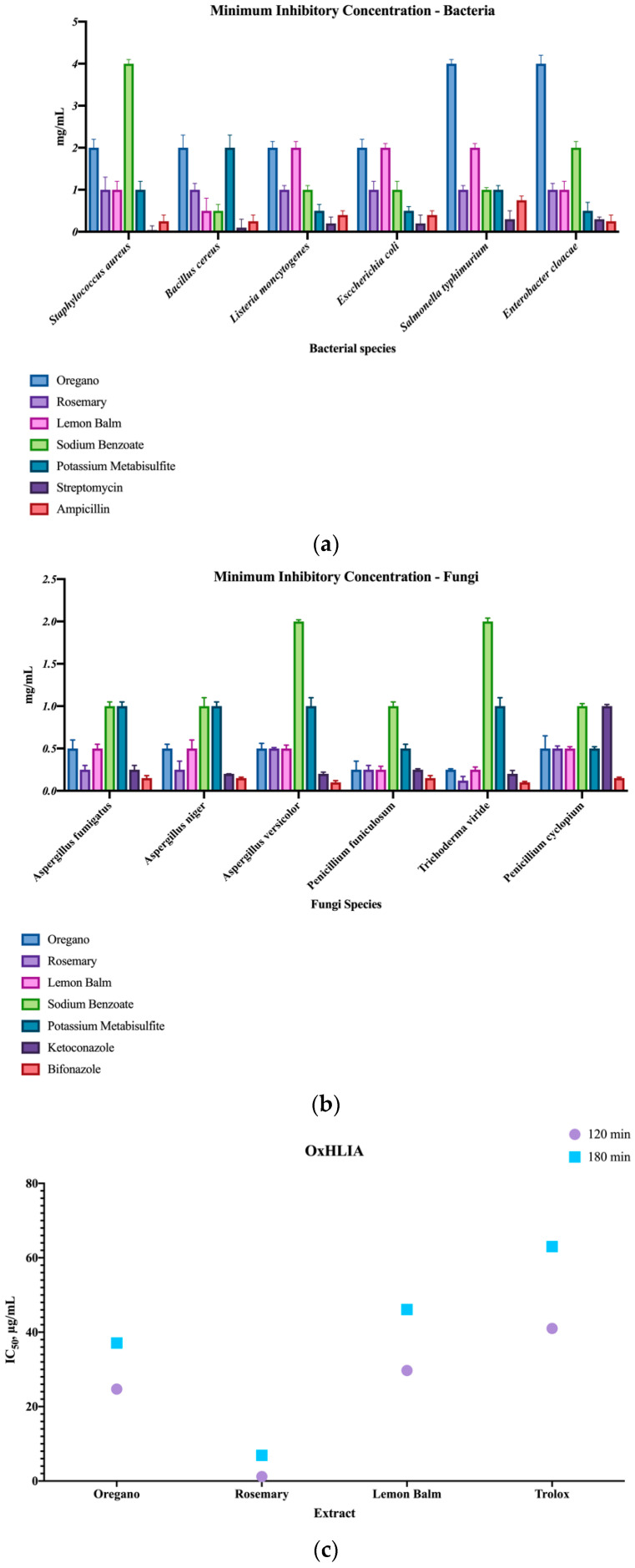
Minimum inhibitory concentrations of bacterial (**a**) and fungi (**b**) strains, as well as the IC_50_ values for the oxidative hemolysis inhibition (OxHLIA) assay (**c**).

The antimicrobial activity was performed on food contaminants, and to compare the effects of the extracts, two artificial preservatives widely used in the food industry, sodium benzoate (E211) and potassium metabisulfite (E224) were also tested, as well as two antibiotics and antifungals that corresponded to the positive controls—streptomycin and ampicillin for the bacteria, and ketoconazole and bifonazole for the fungi. Figure 1a shows the minimum inhibitory concentration (MIC) of six bacterial strains, while Figure 1b shows the MICs for six food fungal contaminants. Analyzing Figure 1a, it is clear that both the natural extracts and the synthetic ones were higher (lower activity) than the positive controls, which is quite expected due to the positive controls being antibiotics, and the extracts and additives, compounds with broad antimicrobial activity. Still, rosemary proved to be the best extract by showing similar effects as sodium benzoate, except for *Bacillus cereus*, where lemon balm showed the best effects, in exequo with sodium benzoate. Figure 1b clearly shows that the plant extracts have a higher inhibition in fungal species than bacterial ones, showing lower MICs than both sodium benzoate and potassium metabisulfite, with rosemary showing the lowest MIC of all extracts for all fungal strains.

The results of the OxHLIA assay (Figure 1c) show two different timestamps, namely 120 and 180 min, for all three extracts and the positive control, trolox. These two timestamps were defined to analyze variations in the ability of the natural extracts to protect the red blood cells from the free radical generated in the in vitro system over time, since plant extracts contain different antioxidants capable of interacting with each other and offering protection at different time periods. All extracts showed a stronger activity, (lower IC_50_ values) than trolox for both timestamps. Among the natural extracts, rosemary showed the biggest potential, with EC_50_ values approximately 20-fold under oregano and 25 under lemon balm. The increase from 120 to 180 min showed, as expected, a significant increase in the extract concentration required to protect the red blood cell population. Even though lemon balm showed the highest amount of phenolic compounds, rosemary was overall the extract with the best bioactivities and preserving capabilities, showing that these may arise from synergistic effect among the bioactive constituents of the extract. Furthermore, this could also be explained by the way the phenolic compounds interact both with the peroxyl radicals (resulting from the thermal decomposition of AAPH) and the red blood cell membranes, as well as by their ability to act in a time-dependent manner. Regarding the cytotoxic analysis, all the extracts revealed no toxicity for normal PLP cells at a maximum of 400 μg/mL, thus showing them to be safe for consumption, although this activity is a screening and in vitro tool, meaning that to ensure the complete safety of the use of these extracts, other in vitro and in vivo studies are needed. Furthermore, it was already expected that these three extracts would not present toxicity, since these plants are usually consumed worldwide.

### 3.3. Muffin Incorporation an Analysis

#### 3.3.1. Nutritional Profile

Table 1 shows the nutritional profile of the muffins preserved with the natural and artificial preservatives in both the ideal concentrations but also at the highest allowed dose within the European Union for potassium sorbate. This table shows the result of a simultaneous analysis of both independent factors, namely storage time (ST) and type of preservative (TP). This allows us to understand the effect of each towards the outcome in each situation, or if there was a significative interaction between both. Thus, the top section of the table, which represents ST, has all the values of the preservatives for each timestamp, while the bottom part has all storage times for each preservative. When a significative interaction is detected between ST and TP (*p*-value < 0.05), some conclusions can be extracted by the estimated marginal means (EMM). Inversely, when the *p*-value > 0.05, post hoc tests are used to classify each factor (Tukey’s test for homoscedastic samples and Tahmane T2 for no homoscedastic ones).

Due to being a food made from flour, the nutritional profile included moisture, ash, fiber, carbohydrates, crude fat and proteins. The highest nutrients were carbohydrates which corresponded to approximately 64 g/100 g fw, followed by moisture and crude fat, which rendered proteins and fiber the least abundant nutrients. In line with what is required from food additives (in which preservatives are included), which should not change the food it is used in, beyond the required effect for its addition, the different extracts in all nutrients did not alter the nutritional profile of the muffin. Only the fibers showed a statistically significative increase from T4 to T8 days, showing that storage time had a greater influence on the nutritional characteristics than the addition of the different extracts. Considering the partial Eta squared, both ST, TP and the interaction in both showed a very similar percentage for variation of these values, thus reinforcing the fact that no factor prevailed over the other. Table 1 also shows the four organic acids detected, namely oxalic, quinic, citric, and succinic acids, as well as the total amount of organic acids. Among these compounds, a significative interaction between ST and TP was found for oxalic acid, while quinic, citric and succinic acid showed statistical differences. For quinic acid, ST showed an influence on its amount, with statistical significative differences from T0 to T4. Inversely, for succinic acid, TP had a relevant influence, showing that this acid was better preserved by potassium sorbate at 2 mg/g, and the least in the control muffin. The total amount of organic acids was also influenced by both factors but in this case, the influence of each one was clearly defined. The decrease was statistically significant from T0 to T8, showing potassium sorbate at 2 mg/g was the extract that best preserved them. Still, this amount is very rarely used in food, and thus, the extracts, with no statistical difference for the usual amount of potassium sorbate, 0.2 mg/g, proved to have the same effect as the artificial preservative. Table 1 also shows the two detected soluble sugars, namely sucrose and maltose. Detection of sucrose was due to the sugar added to the recipe, and maltose is the most abundant sugar in flour. A significative interaction was found for both sugars and for the total sugars, with a partial Eta squared accounting for about 25% of the variance in the interaction of both factors, 15% for the preservative type and not even 10% for time, thus not allowing for any major conclusions.

#### 3.3.2. Fatty Acid Profile

Table 2, formatted in the same way as Table 1, shows the profile of the individual fatty acids. Only the 11 major fatty acids are shown in the table, as the others identified were in very low amounts or trace values. The saturated (SFA), monounsaturated (MUFA), and polyunsaturated (PUFA) fatty acids are also represented, showing higher amounts of PUFA, which varied between 48 and 54%, followed by MUFA and finally SFA, deeming the muffins quite poor in SFA, which are known to be bad for human health, even if the overall amount of fat is considerable. Individually, the most abundant fatty acid was linoleic acid (C18:2), followed by oleic acid (C18:1) with approximately 50 to 55%, and 30 to 33%, respectively. In relation to the contribution of each factor, ST and TP, there was a significant interaction between both factors for all individual fatty acids, SFA, MUFA and PUFA, showing that neither factor showed an influence over the other, which is in line with what is expected from food additives. Furthermore, by analyzing the partial Eta squared of the interaction of the two factors, it was responsible for over 50% of the variation verified in all fatty acids.

#### 3.3.3. Physical Parameters Analysis

The external color of the top and the side of the muffins was also recorded using a portable colorimeter and expressing the values in L*, a* and b* using the 1987 CieLAB. Furthermore, texture profile analysis (TPA) was performed on the muffins to understand its texture and subsequent variation due to the passage of time and incorporation of the extracts. Thus, Table 3 shows these physical analyses, and is also arranged according to the two-way ANOVA. L* represents lightness, a* represents greenness-redness and b* blueness-yellowness. The top of the muffins showed higher values of lightness when compared to the side. For the top section, the influence of ST was clearly understood due to a decrease in L* from 56 to 50 while increasing a* and b* with statistical differences. Adding to these changes resulting from ST, TP also induced some changes, namely the extract of rosemary than showed the least change in L* when compared to the control muffin. a* did not show considerable differences, although lemon balm at the maximum level was the extract that showed the lowest statistical value. For the top section, no considerable differences were sought for b*. The side of the muffins did not show statistically relevant changes in terms of L* due to a significative interaction among both factors. For a* and b*, the extracts showed a higher influence than ST; thus, lemon balm at 2 mg/g showed a greater shift to green (a*), due to the intense green color of the extract. The b* values also showed significant interaction between the two factors not allowing for concrete conclusions.

The middle section of Table 3 shows the different dimensions of texture after TPA analysis of the muffins. Hardness (force necessary to chew the food), adhesiveness (adhesion force between the food and teeth) and springiness (recovery force of the food after deformation) showed statistical differences. For hardness, as expected, an increase was measured over time, with close to no influence from the extracts in this parameter. Inversely, adhesiveness showed a reduction over the same period, which was also expected, for these two dimensions are related. Springiness is the dimension where the influence of both factors is clearer, due to the crumbling of the muffin being the inverse of springiness. Thus, as expected, the springiness reduced over time, with significant difference from T0 to T4, and in relation to the extracts, the control sample showed the least springiness, while rosemary and potassium sorbate incorporated muffins showed the highest springiness, showing their good maintenance of this appreciated quality. Resilience, cohesiveness, gumminess and chewiness showed a significant interaction.

**Figure 2:**
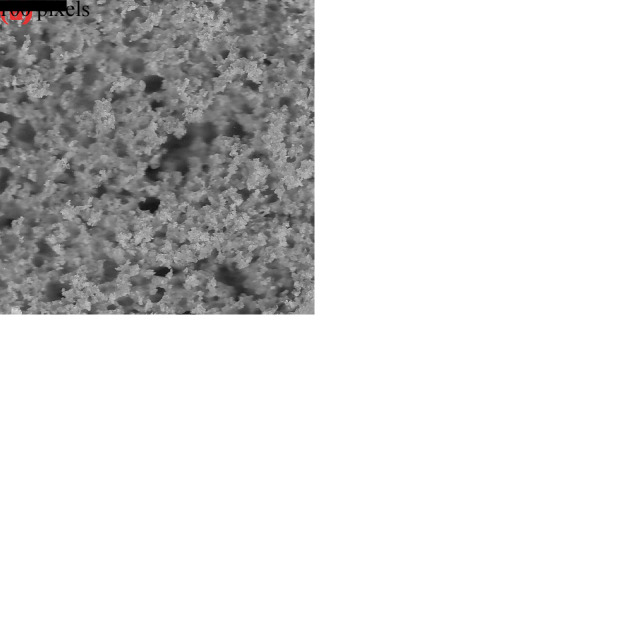
Muffin crumb images from control muffin at 0 days (**a**,**b**), and muffin with rosemary extract at the 8th day of analysis (**c,d**). Images (**b**,**d**) are binary images obtained by using the auto threshold the Otsu algorithm.

Finally, the right-hand section of Table 3 shows the results of some features of the muffin crumb by digital image analysis. The first feature if the number of objects (holes) found in the crumb (Figure 2). The crumb of flour-based foods is influenced by many parameters, and thus, the number of “holes” in the crumb can be influenced by the added extracts and/or the passage of the 8 days of analysis. Thus, the number of objects represents the number of holes found in the crumb of the different muffins, showing that the passage of time had a greater influence than the addition of the extracts, although no statistically significant difference was sought. The average size refers to the average size of the holes in the crumb, measured in squared pixels, show that once again, the extracts did not have a statistically significant influence on the size of the holes, although ST did show an influence. The average size reduced over time, probably due to the migration of water from the crumb to the crust as part of the staling process, thus reducing the size of the holes. Finally, the circularity refers to the roundness of the holes of the crumb, and for this feature, a significant interaction was found for ST and TP, not allowing for conclusions, although there does not seem to be major variation in this parameter.

#### 3.3.4. Linear Discriminant Analysis (LDA)

**Figure 3:**
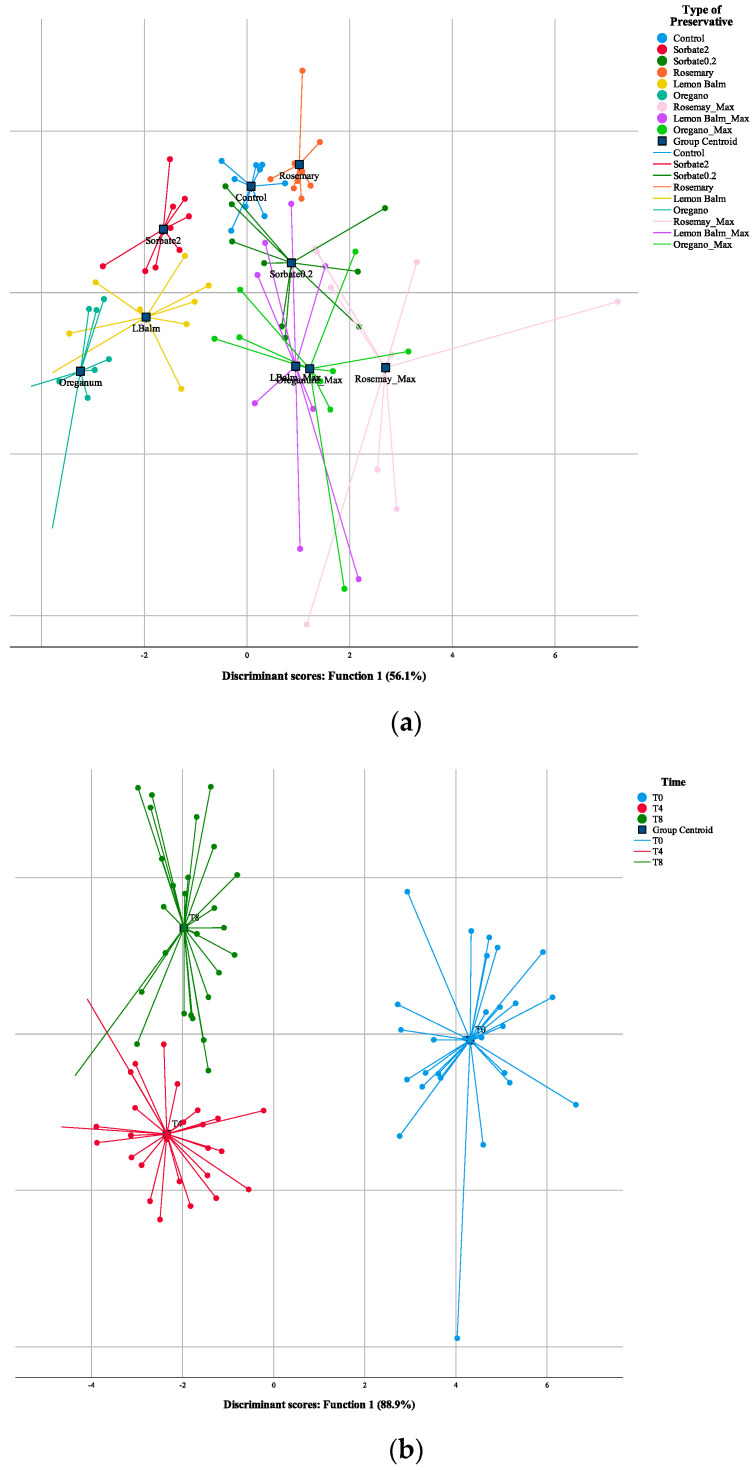
Spatial distribution of type of preservative (TP) (**a**) and storage time (ST) (**b**) markers following the distribution set by the canonical discriminant functions coefficients. For TP, Function 1 accounted for 56.1% of the variation and Function 2 accounted for 17%. Combined, these two functions accounted for 73.1% of the variation, with another 4 functions necessary to account for 100%. Regarding ST, Function 1 accounted for 88.9% of the variation and Function 2 accounted for 11.1%, together accounting for 100% of the variation of the factor.

Despite the very slight differences between the different incorporated muffins, LDA was performed to assess the magnitude of these differences, and to understand if they were enough to discriminate each preservative and storage time (Figure 3). Thus, regarding TP, the model defined six functions that accounted for 100% of the variance (Function 1–56.1%, Function 2–17.0%; Function 3–10.7%; Function 4–9.3%; Function 5–6.6% and Function 6–0.3%) (Figure 3a). Of the 45 analyzed parameters (variables), six showed discriminant ability for TP, namely C18:1 (oleic acid), C16:1 (palmitoleic acid), C16:0 (palmitic acid), L* of the side of the muffins, C18:0 (stearic acid) and citric acid. Of these, those that mostly correlated with Function 1 were palmitoleic acid, oleic acid and L* of the side of the muffins, while palmitoleic acid, stearic acid and L* of the side of the muffin were correlated with Function 2. Observing Figure 3a, it is clear that, based on these the parameters with higher discriminant ability for Function 1 separated the muffins incorporated with Sorbate at 2 mg/g, lemon balm and oregano from the rest of the muffins, which was a considerable discrimination due to Function 1 being responsible for 56.1% of variance. Function 2, which only accounted for 17% of variance, further divided the extracts, although to a lesser extent. Still, a cluster of the three natural extracts at maximum level was seen, meaning the effect of these extracts is quite similar at the concentration of 2 mg/g. Equidistant from sorbate at 0.2 mg/g is the cluster of lemon balm and oregano at the maximum amount, but also rosemary, quite close to the control sample. Considering sorbate at 0.2 mg/g is the usual preservative for these types of muffins, it can be concluded that lemon balm and oregano at maximum amount are the most similar, although rosemary also has similar preserving capacity, showing very little difference from the control sample, thus showing a lower effect on the studied parameters.

Considering ST, from the 45 analyses, the ones with discriminant ability for ST were gomosity, ash, L* of the top of the muffin, circularity, L* of the side of the muffin, C20:1 (eicosanoic acid) and springiness. Of these, those that mostly correlated with Function 1 were the L* of the top of the muffin, the L* of the side of the muffin, and springiness, while ash, eiconsanoic acid, and the L* of the side of the muffin mostly correlated with Function 2. Interestingly, variations in these analyses are related to the passage of time and changes due to stalling, which is a process that preservatives do not undergo. Analyzing Figure 3b, it is clear that based on these parameters, Function 1, accounting for 88.9% of variance, separated T0 from the other two time stamps, namely T4 and T8, while Function 2, which only accounted for 11.1% of variance, separated both these times of 4 and 8 days. To the author’s best knowledge, this is one of the first if not the first report of the incorporation of natural preservatives in chocolate muffins, hence the lack of comparison with other previous reports.

## 4. Conclusions

This work evaluated the preserving effect of natural extracts in chocolate muffins, comparing them to artificial counterparts. By applying an optimization extraction protocol, there was a reduction in the number of necessary extractions steps to find the optimal UAE for rosemary, lemon balm, and oregano. Overall, the extracts showed high antioxidant activity, with rosemary being the most antioxidant of all three extracts, even if it was not the one with the highest quantity of phenolic compounds or rosmarinic acid. Regarding the antimicrobial activity, the extracts showed stronger activity against fungi than bacteria, even better than widely used food preservatives. This is important due to muffins being commonly contaminated with fungi species. Adding to these favorable activities, no toxicity was found for the extracts against primary cell lines, even at the highest concentration tested. After incorporating the muffins, it is quite clear that none of the extracts showed deep changes regarding the nutritional chemical and physical profiles, although there was a change in lightness for the muffins incorporated with the highest doses of the natural extracts. The use of the natural extracts at the maximum level allowed for potassium sorbate did not seem to have any beneficial effect on the preserving capacity. Overall, the LDA allowed us to recognize rosemary as the most promising preservative for chocolate muffins due to its similarity in the overall profile to the control sample and the strong antimicrobial and antioxidant capacity of the extract.

## Figures and Tables

**Table 1:** Nutritional profile (g/100 g fresh weight), energy, organic acids (g/100 g) and soluble sugars (g/100 g) of the muffins incorporated with the different extracts during the 8 days of storage time. In each row, different letters represent statistical significative differences with a significance level of 0.05. The SDs were calculated from results obtained in different conditions, and thus should not be considered as precision measurements but rather as an interval of values.

		Moisture	Crude Fat	Protein	Ash	Total Fibre	Carbohydrates	Energy (kcal)	Energy (kj)	Oxalic Acid	Quinic Acid	Citric Acid	Succinic Acid	Total Organic Acids	Sucrose	Maltose	Total Soluble Sugars
Storage Time (ST)	0 Days	19 ± 2	11 ± 1	1.66 ± 0.06	5 ± 1	3.9 ± 0.1 ^a^	64 ± 2	371 ± 9	1551 ± 40	0.07 ± 0.02	0.83 ± 0.02 ^b^	0.8 ± 0.1	0.07 ± 0.07	1.80 ± 0.06 ^b^	19 ± 3	9 ± 1	27 ± 5
	4 Days	18 ± 1	11.2 ± 0.5	1.8 ± 0.1	2.2 ± 0.4	4.1 ± 0.2 ^a^	64 ± 2	374 ± 7	1567 ± 30	0.07 ± 0.01	0.71 ± 0.02 ^a^	0.78 ± 0.08	0.08 ± 0.06	1.71 ± 0.03 ^a,b^	19 ± 4	9 ± 2	28 ± 6
	8 Days	18.3 ± 0.6	10.5 ± 0.7	1.7 ± 0.1	4 ± 2	4.8 ± 0.2 ^b^	65 ± 1	370 ± 5	1548 ± 20	0.07 ± 0.02	0.71 ± 0.01 ^a^	0.7 ± 0.1	0.07 ± 0.06	1.68 ± 0.02 ^a^	19 ± 4	9 ± 2	28 ± 6
*p*-value (*n* = 3)	Tukey test	0.059	0.004	<0.001	<0.001	0002	0.356	0.027	0.027	0.535	0.016	<0.001	0.749	0.023	0.828	0.884	0.848
Type of Preservative (TP)	Control	18 ± 1	11 ± 2	1.76 ± 0.09	4 ± 1	4.0 ± 0.6	65 ± 3	373 ± 10	1561 ± 43	0.07 ± 0.02	0.8 ± 0.3	0.7 ± 0.1	0.08 ± 0.08 ^a^	1.7 ± 0.3 ^a,b^	17 ± 4	8 ± 2	26 ± 5
	Potassium sorbate 2	19 ± 2	10.9 ± 0.5	1.7 ± 0.1	5 ± 2	4.4 ± 0.6	64 ± 2	369 ± 9	1544 ± 40	0.08 ± 0.01	0.9 ± 0.2	0.9 ± 0.1	0.13 ± 0.03 ^c^	2.0 ± 0.4 ^b^	19 ± 4	9 ± 2	28 ± 6
	Potassium sorbate 0.2	18 ± 1	11.2 ± 0.6	1.8 ± 0.1	4 ± 2	4.4 ± 0.6	65 ± 1	376 ± 6	1574 ± 26	0.08 ± 0.01	0.8 ± 0.2	0.78 ± 0.09	0.05 ± 0.04 ^a–c^	1.7 ± 0.3 ^a,b^	18 ± 4	8 ± 2	26 ± 6
	Rosemary	17.8 ± 0.4	11.1 ± 0.4	1.77 ± 0.09	4 ± 2	3.4 ± 0.9	66 ± 1	377 ± 2	1580 ± 10	0.07 ± 0.02	0.8 ± 0.1	0.8 ± 0.1	0.07 ± 0.05 ^b^	1.8 ± 0.3 ^a,b^	21 ± 3	10 ± 1	32 ± 4
	Rosemary Max	18.3 ± 0.7	10 ± 1	1.64 ± 0.06	4 ± 2	4.4 ± 0.7	65 ± 1	371 ± 7	1551 ± 31	0.07 ± 0.02	0.6 ± 0.2	0.6 ± 0.1	0.05 ± 0.03 ^c^	1.5 ± 0.2 ^a^	20 ± 3	8 ± 2	29 ± 5
	Lemon balm	19 ± 2	10.8 ± 0.6	1.66 ± 0.08	4 ± 2	4.0 ± 0.7	64 ± 2	368 ± 10	1542 ± 41	0.07 ± 0.02	0.7 ± 0.2	0.7 ± 0.1	0.03 ± 0.01 ^a,b^	1.6 ± 0.2 ^a^	18 ± 2	8.5 ± 0.9	26 ± 2
	Lemon balm Max	18.7 ± 0.7	10.9 ± 0.5	1.6 ± 0.1	4 ± 2	4 ± 1	64.5 ± 0.9	371 ± 5	1552 ± 20	0.07 ± 0.01	0.7 ± 0.2	0.9 ± 0.1	0.17 ± 0.08 ^b,c^	1.8 ± 0.3 ^a,b^	20 ± 2	9.6 ± 0.9	30 ± 3
	Oregano	19 ± 1	10.6 ± 0.6	1.68 ± 0.05	4 ± 2	4.1 ± 0.7	65 ± 1	369 ± 8	1544 ± 32	0.07 ± 0.02	0.7 ± 0.2	0.76 ± 0.07	0.06 ± 0.04 ^a–c^	1.6 ± 0.2 ^a,b^	16 ± 6	8 ± 3	24 ± 9
	Oregano Max	18.9 ± 0.7	11.1 ± 0.3	1.7 ± 0.2	2.4 ± 0.6	4.5 ± 0.9	63.8 ± 0.9	371 ± 4	1552 ± 17	0.06 ± 0.01	0.7 ± 0.2	0.74 ± 0.04	0.10 ± 0.04 ^a^^,b^	1.6 ± 0.1 ^a,b^	20 ± 3	10 ± 1	30 ± 4
*p*-value (*n* = 9)	Tukey test	0.052	0.745	0.001	<0.001	0.055	0.119	0.030	0.030	0.093	0.189	<0.001	0.062	0.014	0.407	0.253	0.353
ST×TP (*n* = 27)	*p*-value	0.072	<0.001	<0.001	<0.001	0.150	0.140	0.012	0.012	0.038	0.225	0.082	0.313	0.423	0.316	0.289	0.308

**Table 2:** Individual fatty acids profile, expressed in relative percentage, of the muffins incorporated with the different extracts during the 8 days of storage time.

		C14:0	C16:0	C16:1	C18:0	C18:1	C18:2	C18:3	C20:0	C20:1	C22:0	C24:0	SFA	MUFA	PUFA
Storage Time (ST)	0 Days	0.3 ± 0.2	10 ± 1	0.36 ± 0.09	5.0 ± 0.8	33 ± 4	50 ± 5	0.28 ± 0.06	0.4 ± 0.1	0.23 ± 0.04	0.9 ± 0.2	0.5 ± 0.3	17 ± 2	33 ± 4	50 ± 4
	4 Days	0.16 ± 0.02	9.2 ± 0.8	0.32 ± 0.05	4 ± 1	33 ± 5	51 ± 5	0.24 ± 0.04	0.31 ± 0.03	0.20 ± 0.02	0.7 ± 0.1	0.30 ± 0.08	15 ± 2	34 ± 6	51 ± 5
	8 Days	0.16 ± 0.04	10 ± 1	0.35 ± 0.08	5.2 ± 0.2	31 ± 1	51 ± 3	0.27 ± 0.03	0.33 ± 0.02	0.22 ± 0.01	0.70 ± 0.09	0.26 ± 0.05	16 ± 2	32 ± 1	52 ± 3
*p*-value (*n* = 3)	Tukey test	<0.001	0.001	0.001	<0.001	0.001	0.008	0.016	0.006	<0.001	<0.001	<0.001	<0.001	<0.001	0.063
Type of Preservative (TP)	Control	0.3 ± 0.2	10.3 ± 0.4	0.37 ± 0.01	5.3 ± 0.2	30.2 ± 0.4	51 ± 1	0.30 ± 0.02	0.32 ± 0.03	0.21 ± 0.01	0.7 ± 0.1	0.3 ± 0.1	17.3 ± 0.8	30.8 ± 0.3	51.9 ± 0.9
	Potassium sorbate 2	0.2 ± 0.2	8.8 ± 0.7	0.28 ± 0.03	5.2 ± 0.1	30.3 ± 0.4	53.0 ± 0.7	0.24 ± 0.01	0.4 ± 0.2	0.23 ± 0.04	0.9 ± 0.4	0.5 ± 0.5	16.0 ± 0.8	30.8 ± 0.4	53.2 ± 0.8
	Potassium sorbate 0.2	0.18 ± 0.04	11 ± 2	0.39 ± 0.09	5.3 ± 0.3	31 ± 1	50 ± 4	0.31 ± 0.05	0.33 ± 0.03	0.22 ± 0.01	0.82 ± 0.09	0.34 ± 0.08	17 ± 2	32 ± 2	50 ± 4
	Rosemary	0.17 ± 0.03	10.0 ± 0.5	0.38 ± 0.02	5.1 ± 0.1	30.0 ± 0.2	52.4 ± 0.4	0.27 ± 0.02	0.32 ± 0.03	0.22 ± 0.02	0.7 ± 0.1	0.34 ± 0.08	16.7 ± 0.5	30.6 ± 0.6	52.6 ± 0.4
	Rosemary Max	0.17 ± 0.02	10 ± 1	0.41 ± 0.07	4.7 ± 0.6	38 ± 6	44 ± 6	0.24 ± 0.03	0.31 ± 0.04	0.21 ± 0.03	0.74 ± 0.09	0.31 ± 0.05	17 ± 2	39 ± 6	45 ± 6
	Lemon balm	0.2 ± 0.2	9.5 ± 0.7	0.29 ± 0.01	5.4 ± 0.3	29.8 ± 0.5	53 ± 1	0.29 ± 0.05	0.34 ± 0.04	0.24 ± 0.03	0.7 ± 0.1	0.28 ± 0.08	16 ± 1	30.4 ± 0.5	53 ± 1
	Lemon balm Max	0.16 ± 0.03	9 ± 1	0.35 ± 0.06	4 ± 1	37 ± 5	47 ± 5	0.24 ± 0.03	0.31 ± 0.04	0.21 ± 0.02	0.70 ± 0.08	0.29 ± 0.05	16 ± 3	37 ± 5	48 ± 5
	Oregano	0.15 ± 0.04	8.6 ± 0.6	0.23 ± 0.06	4.9 ± 0.1	30.3 ± 0.5	53.8 ± 0.5	0.26 ± 0.06	0.34 ± 0.02	0.21 ± 0.02	0.84 ± 0.08	0.35 ± 0.06	15.3 ± 0.8	30.7 ± 0.4	54.0 ± 0.5
	Oregano Max	0.16 ± 0.04	10 ± 2	0.37 ± 0.08	4 ± 1	35 ± 2	49 ± 2	0.25 ± 0.05	0.31 ± 0.05	0.21 ± 0.03	0.70 ± 0.09	0.30 ± 0.06	15 ± 3	35 ± 2	50 ± 2
*p*-value (*n* = 9)	Tukey test	<0.001	<0.001	<0.001	<0.001	<0.001	<0.001	<0.001	0.028	0.004	<0.001	<0.001	0.002	<0.001	<0.001
ST×TP (*n* = 27)	*p*-value	<0.001	<0.001	<0.001	0.010	<0.001	<0.001	0.005	<0.001	<0.001	<0.001	<0.001	<0.001	<0.001	<0.001

The SD were calculated from results obtained in different conditions, and thus should not be considered as precision measurement but rather an interval of values. SFA—saturated fatty acids; MUFA—monounsaturated fatty acids; PUFA—polyunsaturated fatty acids.

**Table 3:** External color (top and side), different dimensions of texture, and features of the crumb through digital imaging of the muffins incorporated with the different extracts during the 8 days of storage time.

		Top			Side			Hardness (g)	Adhesivity (g/s)	Resilience (%)	Cohesiveness	Springiness (%)	Gumminess	Chewiness	Number of Objects	Average Size	Circularity
		L*	A*	B*	L*	a*	b*										
Storage Time (ST)	0 Days	56 ± 1 ^b^	10 ± 1 ^a^	26.4 ± 0.3 ^a^	45 ± 3	15 ± 4	31 ± 4	1523 ± 1956 ^a^	−1.8 ± 0.2 ^b^	19 ± 3	0.50 ± 0.06	85 ± 3 ^b^	548 ± 247	424 ± 247	97 ± 32 ^a^	23,656 ± 3475 ^b^	38 ± 3
	4 Days	50 ± 1 ^a^	13 ± 1 ^b^	28 ± 1 ^b^	45 ± 3	14 ± 2	32 ± 3	2533 ± 405 ^a,b^	−0.4 ± 0.3 ^a,b^	19 ± 2	0.51 ± 0.03	80 ± 4 ^a^	1292 ± 214	1042 ± 179	96 ± 20 ^a^	18,338 ± 1295 ª	38 ± 2
	8 Days	50 ± 1 ^a^	13 ± 1 ^b^	28 ± 1 ^b^	46 ± 3	14 ± 2	32 ± 3	3599 ± 4287 ^b^	−0.5 ± 0.9 ^a^	18 ± 1	0.50 ± 0.03	80 ± 3 ^a^	1366 ± 267	1068 ± 232	96 ± 12 ^a^	16,993 ± 1214 ª	36 ± 2
*p*-value (*n* = 3)	Tukey test	<0.001	<0.001	0.025	0.023	0.153	0.211	0.026	0.035	0.075	0.324	<0.001	<0.001	<0.001	0.001	0.025	0.358
Type of Preservative (TP)	Control	54 ± 3 ^d^	12 ± 1 ^b–d^	28 ± 3 ^a,b^	50 ± 2	17 ± 1 ^b^	31 ± 6	3116 ± 591	−0.3 ± 0.5	17 ± 1	0.47 ± 0.03	78 ± 4 ^a^	1491 ± 355	1156 ± 266	119 ± 27	18,621 ± 6087	37 ± 3
	Potassium sorbate 2	52 ± 2 ^a–d^	13 ± 1 ^c,d^	29 ± 2 ^a,b^	46 ± 2	16 ± 1 ^b^	33 ± 2	2272 ± 749	−0.7 ± 1.5	18 ± 3	0.50 ± 0.07	83 ± 2 ^b^	1129 ± 558	890 ± 531	112 ± 18	21,213 ± 3236	39 ± 3
	Potassium sorbate 0.2	52 ± 3 ^b–d^	13 ± 2 ^d^	29 ± 2 ^b^	44 ± 5	16 ± 2 ^b^	33 ± 2	1941 ± 1124	−0.2 ± 0.2	19 ± 3	0.51 ± 0.08	83 ± 3 ^b^	992 ± 582	819 ± 475	93 ± 22	21,982 ± 11870	38 ± 2
	Rosemary	54 ± 4 ^d^	12 ± 2 ^b^	27 ± 3 ^a,b^	45 ± 2	15 ± 2 ^a,b^	32 ± 4	1999 ± 763	−0.3 ± 0.2	19 ± 2	0.51 ± 0.03	81 ± 3 ^b^	1053 ± 531	832 ± 490	107 ± 15	17,934 ± 2758	37 ± 2
	Rosemary Max	48 ± 1 ^a^	12 ± 1 ^b,c^	26.3 ± 0.8 ^a^	46 ± 2	13.1 ± 0.7 ^a,b^	31 ± 1	4690 ± 7620	−0.5 ± 0.2	18 ± 2	0.50 ± 0.02	83 ± 3 ^a,b^	1097 ± 424	856 ± 424	97 ± 13	19,088 ± 3513	38 ± 2
	Lemon balm	49 ± 2 ^a–d^	13 ± 2 ^c,d^	29.4 ± 0.7 ^a,b^	44 ± 2	15 ± 2 ^a,b^	33 ± 2	3201 ± 2980	−0.5 ± 0.4	20 ± 2	0.52 ± 0.02	83 ± 3 ^b^	865 ± 312	830 ± 290	88 ± 26	18,392 ± 7376	38 ± 2
	Lemon balm Max	48 ± 2 ^a–c^	11.0 ± 0.4 ^a^	27 ± 1 ^a,b^	45 ± 2	12 ± 1 ^a^	30 ± 2	1901 ± 766	−0.2 ± 0.3	18 ± 2	0.48 ± 0.04	81 ± 4 ^a,b^	865 ± 312	683 ± 236	83 ± 19	19,769 ± 6358	36 ± 4
	Oregano	49 ± 1 ^a,b^	13.4 ± 0.3 ^b,c,d^	27.1 ± 0.7 ^a^	45 ± 3	14 ± 1 ^a,b^	31 ± 2	1998 ± 762	−0.4 ± 0.2	20 ± 2	0.51 ± 0.03	83 ± 4 ^b^	1008 ± 177	830 ± 290	83 ± 16	19,769 ± 6358	37 ± 3
	Oregano Max	51.3 ± 0.6 ^c,d^	12.8 ± 0.1 ^b^	28.3 ± 0.5 ^a,b^	47 ± 1	13.4 ± 0.9 ^a,b^	30 ± 2	1846 ± 717	−0.3 ± 0.2	19 ± 1	0.50 ± 0.02	81 ± 3 ^a,b^	975 ± 423	719 ± 243	86 ± 16	20,677 ± 7740	36 ± 3
*p*-value (*n* = 9)	Tukey test	<0.001	<0.001	0.003	<0.001	<0.001	0.382	0.375	0.579	0.003	0.066	0.001	<0.001	<0.001	0.438	0.065	0.256
ST × TP (*n* = 27)	*p*-value	0.251	0.310	0.558	<0.001	0.304	0.812	0.505	0.249	<0.001	0.001	0.108	<0.001	<0.001	0.412	0.185	0.589

In each row, different letters represent statistical significative differences with a significance level of 0.05. The SDs were calculated from results obtained in different conditions, and thus should not be considered as precision measurements but rather as an interval of values.

## Data Availability

Data available on request.
